# Methylation associated transcriptional repression of *ELOVL5* in novel colorectal cancer cell lines

**DOI:** 10.1371/journal.pone.0184900

**Published:** 2017-09-20

**Authors:** Arnoud Boot, Jan Oosting, Jaap D. H. van Eendenburg, Peter J. K. Kuppen, Hans Morreau, Tom van Wezel

**Affiliations:** 1 Department of Pathology, Leiden University Medical Center, Leiden, The Netherlands; 2 Department of Surgery, Leiden University Medical Center, Leiden, The Netherlands; Sapporo Ika Daigaku, JAPAN

## Abstract

Genetic and epigenetic alterations mark colorectal cancer (CRC). Global hypomethylation is observed in nearly all CRC, but a distinct subset of CRC show the CpG Island Methylator Phenotype (CIMP). These tumors show DNA hypermethylation of a specific subset of CpG islands, resulting in transcriptional downregulation of nearby genes. Recently we reported the establishment of novel CRC cell lines derived from primary and metastatic CRC tissues. In this study we describe the DNA methylation profiling of these low passage CRC cell lines. We generated global DNA methylation profiles with Infinium HumanMethylation450 BeadChips and analysed them in conjunction with matching gene expression profiles. Multidimensional scaling of the DNA methylation and gene expression datasets showed that *BRAF* mutated cell lines form a distinct group. In this group we investigated the 706 loci which we have previously identified to be hypermethylated in *BRAF* mutant CRC. We validated the significant findings in the The Cancer Genome Atlas colon adenocarcinoma dataset. Our analysis identified *ELOVL5*, *FAM127B*, *MTERF1*, *ZNF606* to be subject to transcriptional downregulation through DNA hypermethylation in CRC. We further investigated *ELOVL5* with qPCR and immunohistochemical staining, validating our results, but did not find a clear relation between ELOVL5 expression and tumor stage or relapse free survival. *ELOVL5*, *FAM127B*, *MTERF1*, *ZNF606* are involved in important cellular processes such as apoptosis, lipogenesis and the downstream transcriptional effect of the MAPK-pathway. We have identified a DNA methylation profile regulating key cellular processes in CRC, resulting in a growth advantage to the tumor cells.

## Background

Methylation of the C-5 carbon occurs at approximately 70–80% of all CpG dinucleotides in the human genome [1] and is associated with heterochromatin, a closed chromatin conformation. Heterochromatin at intergenic regions protects genomic stability by inactivation of transposable elements such as LINEs and SINEs. At gene regulatory elements the heterochromatin state inhibitis gene expression by preventing the binding of transcription factors and transcription machinery. Addition of a methyl group to an individual CpG site can disrupt binding of transcription factors or chromatin remodelers [2].

Approximately 70% of all gene promoters contain CpG-islands (CpGI), loci with an elevated CG content. In normal tissues CpGI are generally unmethylated and often serve as sites of transcription initiation, including those located outside of promoter regions [3]. During tumorigenesis, many tumor types show a CpG island methylator phenotype (CIMP) [4].

CIMP is considered one of the major tumorigenesis pathways in colorectal cancer (CRC), together with chromosomal instability, and microsatellite instability [4]. CIMP is associated with tumors from the proximal colon, older age and is more often identified in female patients [5]. Moreover, hypermethylation of CIMP-genes *NEUROG1* and *CDKN2A* is negatively associated with survival [6].

**Fig 1:**
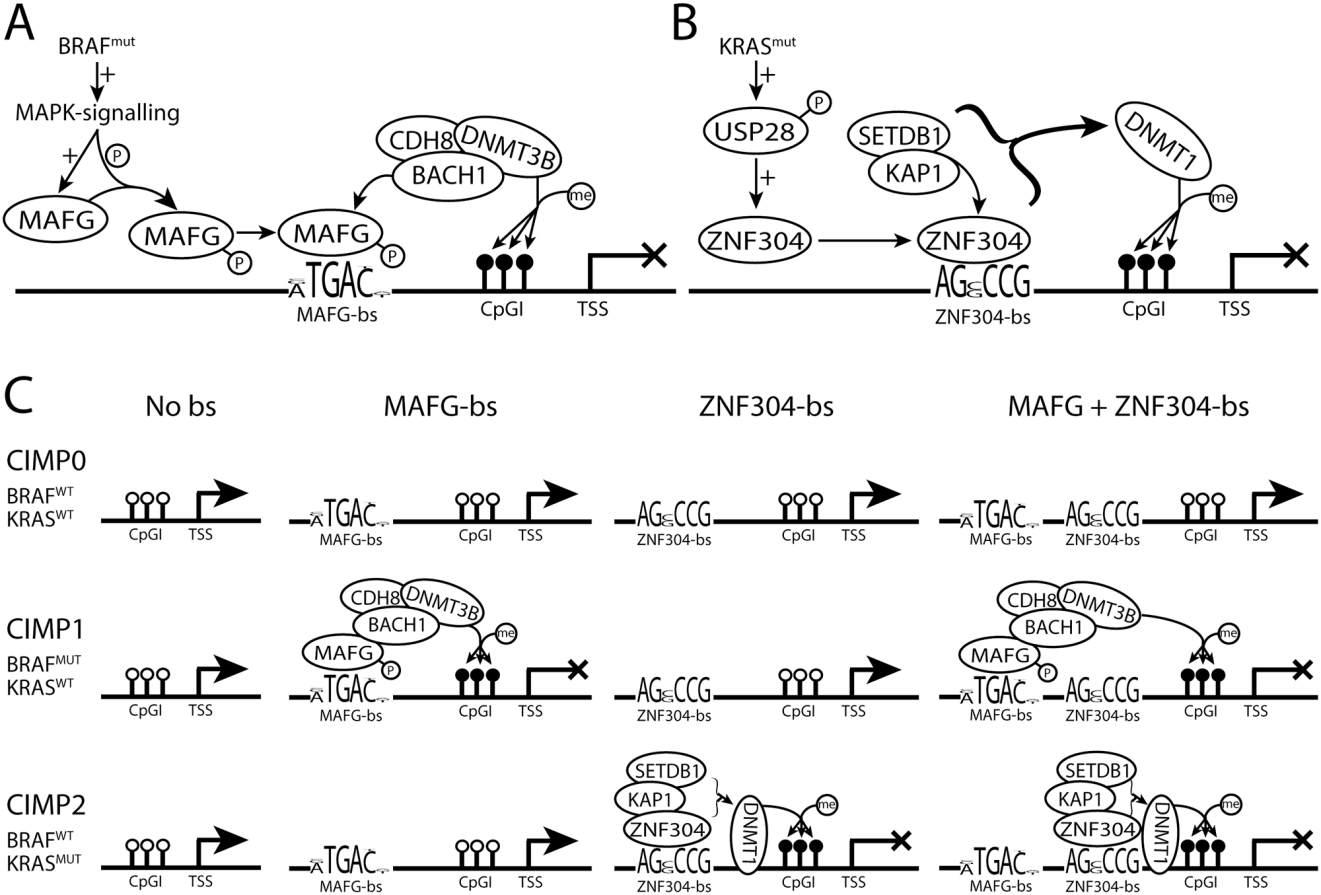
Regulation and targeting of CIMP profiles by MAFG and ZNF304. Mutant KRAS upregulates of deubiquitinase USP28, leading to ZNF304 upregulation. ZNF304 recruits a corepressor complex which subsequently methylates the nearby CpGI. 1B: MAFG is upregulated and phosphorylated through MAPK-signalling which when bound to it’s binding site recruits a corepressor complex resulting in DNA hypermethylation (1B). Overview of DNA methylation outcomes for genes with and without MAFG and ZNF304 binding sites in CIMP0, CIMP1 and CIMP2 tumors (1C). bs = binding site, TSS = Transcription Start Site, CpGI = CpG Island.

CIMP in CRC is tightly associated with the occurrence of *BRAF* mutation. Recent work by Fang et al. proposes a direct relationship between the *BRAF* c.1799T>A mutation, *MAFG* activation and hypermethylation of CIMP genes such as *MLH1*, *NEUROG1* and *CDKN2A* (Fig 1A) [7]. Also *KRAS* mutated tumors show hypermethylation at a large number of CpGI. These CpGI are in part distinct from the hypermethylated CpGI found in *BRAF* mutant tumors. This *KRAS*-mutant associated CIMP profile is also referred to as CIMP-2 [8–10]. *ZNF304* gene expression was identified as a crucial factor for CIMP-2 in *KRAS* mutated CRC (Fig 1B) [10]. Fig 1C contains a graphical representation of the DNA methylation state of CpG islands containing MAFG and ZNF304 binding sites in tumors with and without *BRAF* and *KRAS* mutations. This model suggests a direct causal link between the occurrence of *BRAF* and *KRAS* mutations with specific DNA methylation changes.

We recently described somatic mutation and copy number analysis, chemosensitivity profiling and *MLH1*, *MGMT* and *CDKN2A* methylation status for novel, low passage CRC cell lines. Here we report an integrated genome wide DNA methylation and gene expression profiling of these cell lines using Infinium HumanMethylation450 BeadChips. Multidimensional scaling of the DNA methylation data showed that the *BRAF* mutant cell lines form a distinct group of cell lines. In this group we integrated methylation and gene expression of previously identified loci that are hypermethylated in *BRAF* mutant CRC. The findings were validated using the publically available TCGA colon adenocarcinoma data. Our results revealed an expression signature of 4 genes; *ELOVL5*, *FAM127B*, *MTERF1* and *ZNF606* which is regulated through DNA hypermethylation of their respective promoters.

## Methods

### Cohorts

21 cell lines were studied; a previously published panel of 20 previously described cell lines complemented with cell line JVE017. JVE017 was established from a microsatellite stable cecum adenocarcinoma from a 67 year old female. JVE017 carries mutations in *BRAF* (p.V600E), and *TP53* (c.848G>C; p.R283P) and a homozygous *SMAD4* deletion. JVE017 is partial hypermethylated at the *CDKN2A* promoter and unmethylated at the *MLH1* and *MGMT* promoters.

Anonimised snap frozen tissue samples were collected from the biobank of the Pathology department Leiden University Medical Center (Leiden, The Netherlands). Surgical specimens were collected between 1986 and 2013. Samples were selected on the availability of both carcinoma and normal fresh frozen tissue. Polyp samples were selected based on availability.

The present study was approved by the Medical Ethics committee of the Leiden University Medical Center (protocol P01-019) and analyzed according to the Code for Adequate Secondary Use of Data and Tissue, provided by the Federation of Dutch Medical Scientific Societies (www.federa.org).

Histological examination of the samples was performed prior to nucleic acid isolation. Normal samples were selected to have at least 70% colon epithelium, without any neoplastic cells present. Polyp and carcinoma samples were selected to have at least 70% neoplastic cells. After sectioning for isolations, histological examination was repeated to check these same parameters.

### TMA

We stained a subset of TMA series of 999 colorectal cancer tissues from patients who underwent surgical resection between 2002 and 2008 at the Leiden university medical center [11]. In total 263 tissues were analyzed for which all 3 replicates were measured and consistent.

### DNA isolation

DNA isolation was performed from cells at 70–80% confluency, using the Wizard Genomic DNA Purification Kit (Promega, Madison, WI, USA) according to the manufacturer’s instructions.

### Infinium HumanMethylation450 BeadChips

Infinium HumanMethylation450 BeadChips were processed by ServiceXS (ServiceXS B.V., Leiden, The Netherlands) according to manufacterors description. Data analysis was performed in R version 3.2.1. Raw array data was loaded using the methylumi package [12]. Sites with low beadcount or high detection p-values were removed: 1,370 sites were removed as beadcount <3 in 5% of samples, 5118 sites having 1% of samples with a detection p-value greater than 0.05 were removed. Data was normalized using the BMIQ function included in the wateRmelon package [13]. Sites with a mean total intensity (green + red intensity) below 2,000 were excluded to reduce a possible effect of background signal. Raw data (IDAT-files) and pre-processed data are available through GEO under GSE67775.

### TCGA COAD methylation data

All available Infinium HumanMethylation450 BeadChip data of the colon adenocarcinoma (COAD) study of The Cancer Genome Atlas (TCGA) with known *BRAF* and *KRAS* mutation status was downloaded [14]. IDAT files were processed as described above for the cell line samples. A list of TCGA samples included is shown in S1 Table, including *BRAF* and *KRAS* mutation status.

### Bisulfite sequencing analysis

Bisulfite conversion was performed on 200 ng of DNA using the EZ DNA methylation Gold kit and eluted in 15μL MQ water. The PCR reaction contained 1x IQ SYBR green supermix (#170–8862, Biorad), 1μL of the bisulfite converted DNA and 5 nmol forward and reverse primer. PCR products were purified using the MinElute^®^ 96 UF PCR Purification kit (#28051, Qiagen) and Sanger sequencied at Macrogen (Macrogen Europe). Sequence alignment and quantification of methylation was performed using ESME (Epigenomics Inc.). The primers used were: *CD1d*_BSA_2_Fw: TGTAAAACGACGGCCAGTGTTTAGTTTTAGTTTTTATTGT and *CD1d*_BSA_2_Rev: CAGGAAACAGCTATGACCATAATAACTCTCTTACCTCT.

### qRT-PCR

RNA was isolated from cell cultures at 70–80% confluence. For all samples RNA was isolated using Nucleospin^®^ RNA columns (Machery-Nagel) according to the manufacturers specifications. RNA concentration was determined using a Nanodrop^®^ 1000 (Thermo Scientific). RNA integrity was determined using the Agilent 2100 Bioanalyzer RNA 6000 Nano kit (Agilent Technologies Inc., Santa Clara, CA, USA). Samples with a RIN score < 7.0 were excluded.

cDNA synthesis and qRT-PCR were performed as described previously [15]. Gene expression values were normalized for cDNA input using *CPSF6* and *HNRNPM* as housekeeping genes [15]. For patient samples all expression values displayed are relative to the median of the normal samples. *CD1D* qRT-PCR primers: q*CD1d*_Fw2: GAGCAACCCTGGATGTGGT and q*CD1D*_Rev2: TCAAGCCCATGGAGGTGTA.

### Immunohistochemistry

4 μm sections of the CRC tissue micro array (TMA) were deparaffinized and rehydrated, followed by blocking of endogenous peroxidases by incubating the sections in a 0.3% hydrogen peroxide solution for 20 min (30% hydrogen peroxide, 100x diluted in water). For antigen retrieval the slices were placed in 800 ml preheated Citrate buffer (ph 6) and boiled for 10 min. 100 μl diluted (1: 1000) anti-ELOVL5 (Sigma life sciences, HPA047752) was added per slide and incubated overnight at 4°C. After a 3 x 5 min wash with PBS buffer 100 μl poly-HRP (ILimmunologic, DPVO110HRP) was added and incubated at room temperature for 30 minutes. Then washed 3 x 5 min with PBS. 4 drops of DAB (dilution 1:50) was added to the slides. After 5 minutes the slides were washed in demineralized water, counterstained with haematoxylin, dehydrated and mounted (LEICA, 046430011). Staining of epithelial cells in the tissues was scored as low, medium or high.

### Statistics

Comparisons were performed using the limma package [16]. Multiple testing correction was performed using the Benjamini-Hochberg method.

## Results

### Genome-wide DNA methylation profiling

**Fig 2:**
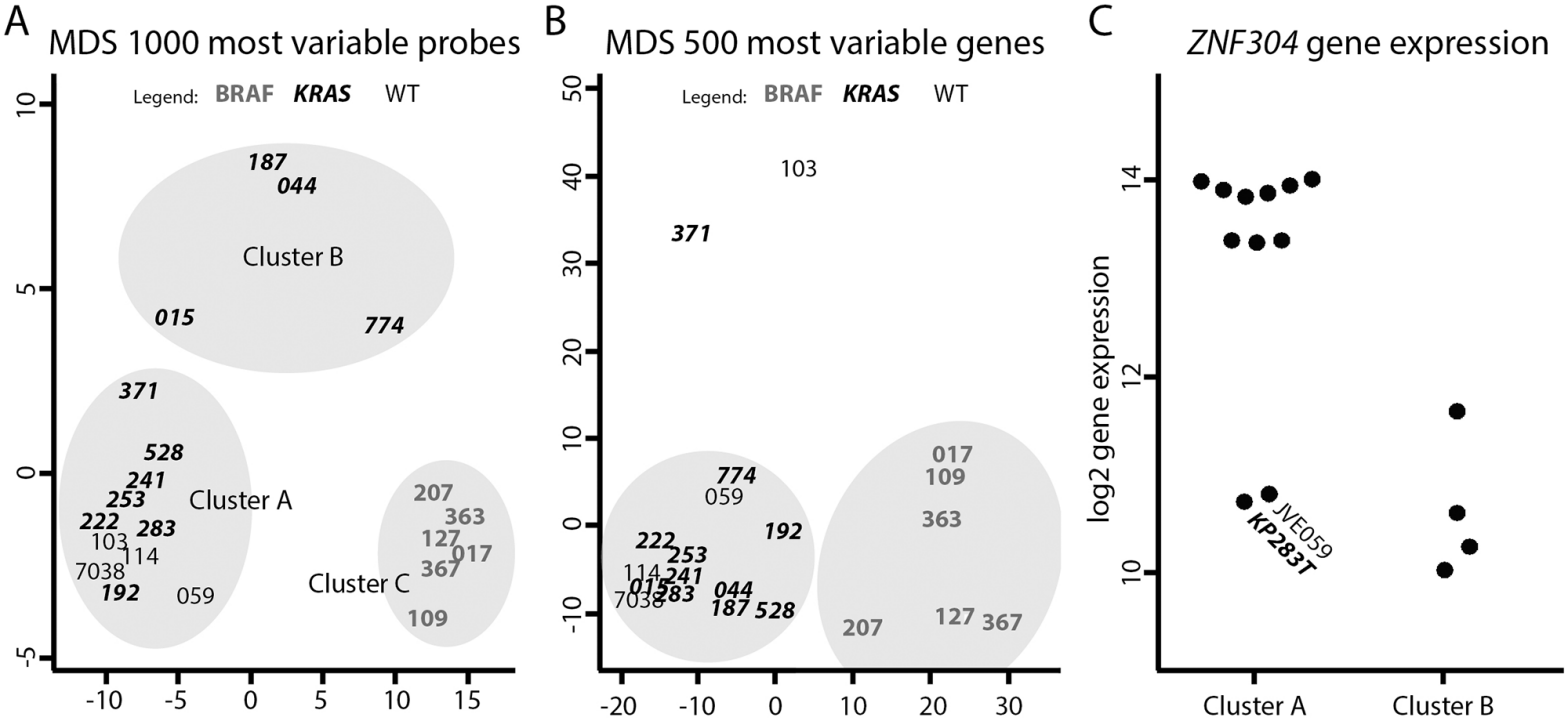
Clustering of gene expression and DNA methylation data. To minimize overlap between cell line names, only the numbers corresponding to the cell lines were displayed. MDS analysis of DNA methylation data shows a distinct cluster of *BRAF* mutant cell lines; cluster C. Secondary clustering is formed by *KRAS* mutant samples with low *ZNF304* expression (cluster B, Figure 2A). MDS analysis of gene expression data showed *BRAF* mutant samples cluster separately from *KRAS* mutant and WT samples (2B). The cell lines in cluster B show a 5.9-fold lower *ZNF304* expression compared to the cell lines in cluster A (2C).

To investigate differential methylation patterns DNA of 21 CRC cell lines was successfully hybridised to Infinium HumanMethylation450 BeadChips. In the initial analysis we performed multidimensional scaling (MDS) of the 1000 most variable methylation loci. In the resulting cluster plot, the six *BRAF*-mutated cell lines formed a distinct subgroup (Fig 2A, Cluster C). Interestingly, when performing MDS on the gene expression dataset, we also found the BRAF-mutant cell lines to form a distinct cluster (Fig 2B).

We observed another distinct cluster (Cluster B) in the DNA methylation MDS plot. Cluster B consists of the 4 cell lines JVE015, JVE044, JVE187 and JVE774. These lines all carry a mutation in *KRAS*. For *KRAS* mutant CRC an association with CIMP-2 status and a high RNA expression of *ZNF304* is described [10]. We decided to investigate the expression of *ZNF304* in the cell lines. Unexpectedly, the cell lines in cluster B showed significantly lower expression of *ZNF304*, on average 5.9-fold lower when compared to the other cell lines (p = 1.9×10^−3^, Fig 2C), which suggests that these cell lines are CIMP0. Contrastingly, when we extract the methylation levels at CIMP-marker loci this indicates that Cluster B is in fact CIMP2-positive and that Cluster A is CIMP0, although we should note that some CIMP markers might be skewed in cell lines, as was previously described for *CDKN2A* [17].

### *BRAF* mutation associated DNA methylation patterns

We previously described the DNA methylation patterns associated with *BRAF* mutations in primary CRC tissues [15]. All 758 differentially methylated loci in *BRAF*-mutated primary tumor samples from this study were analysed in the cell line DNA methylation dataset. 706 of the 758 loci were covered by at least one probe in the 450k DNA methylation dataset when we identified the loci based on the same chromosomal positions. For loci with multiple probes, the average of all probes per sample was taken. When we compared methylation levels between *BRAF* mutant and *BRAF* wildtype cell lines, 338 loci (47.9%) were associated with *BRAF* mutation status. 98.8% of these loci were hypermethylated in the *BRAF* mutant cell lines, which is comparable to our earlier results of 96.3% hypermethylation in *BRAF* mutant samples [15].

Upon closer examination of the loci that were not significantly hypermethylated 52.4% showed a very low variability (standard deviation < 0.2) amongst the cell lines (against 9.2% in the loci with differential methylation). As expected, plotting the variability against the average β-values the non-significant loci, we found low variance at both high and low methylation loci (S1 Fig).

Subsequently, the 338 regions that associated with *BRAF* mutation status were studied. 273 locate in or near known genes or transcripts and of these 220 unique genes are potentially regulated by this DNA methylation. These genes were further studied for RNA expression.

### *BRAF*-associated DNA methylation and gene expression

**Fig 3:**
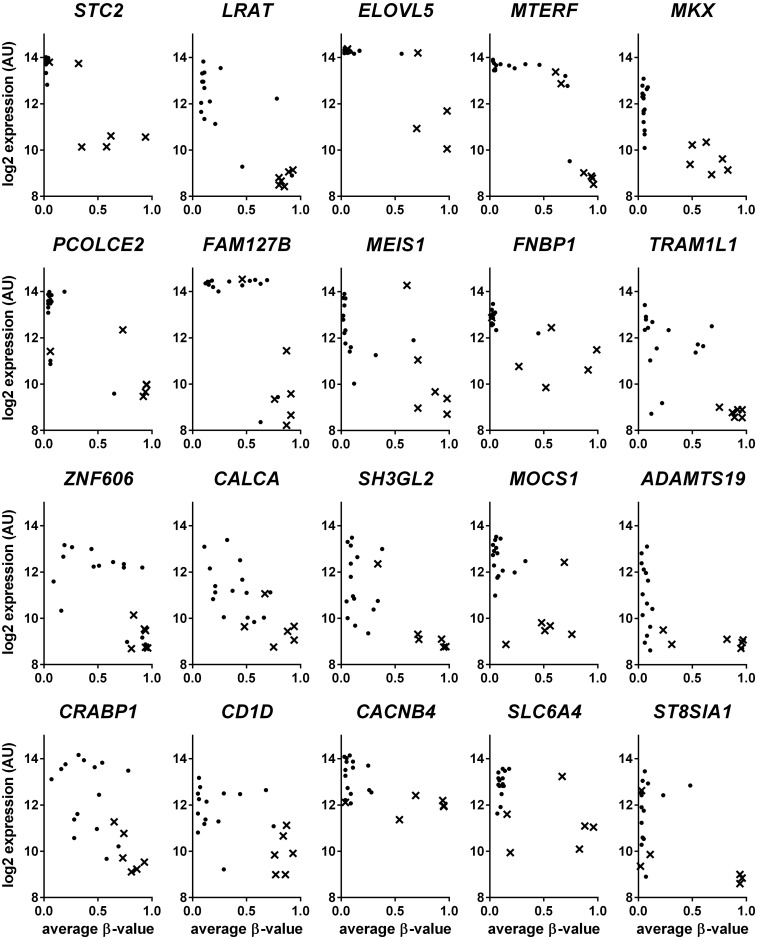
Genes showing a negative correlation between gene expression and DNA methylation. Average β-values are plotted on the x-axis, log2 gene expression values on the y-axis. Dots represent a cell line; *BRAF* mutant cell lines are marked with an x.

In the previously described genome wide expressiondata from the cell lines, 169 of the 220 genes potentially regulated by *BRAF*-associated DNA methylation changes were present. Of these, 31 genes, showed differential gene expression in *BRAF* mutant compared to *BRAF* wildtype cell lines. These 31 genes are linked to 46 differentially methylated genomic regions. We calculated the correlation between gene expression and DNA methylation data and 28 gene-methylation locus pairs were found to be negatively correlated (r < -0.5). These represent a total of 20 unique genes for which the expression was plotted against the corresponding methylation loci (Fig 3). These genes include *LRAT*, *MEIS1*, *ST8SIA1* and *STC2* which have all previously been shown to be subject to transcriptional downregulation as an effect of DNA hypermethylation in human cancers [18–21].

### TCGA methylation data

We validated our findings using the Infinium HumanMethylation450 BeadChip data of the colon adenocarcinoma (COAD dataset of The Cancer Genome Atlas; TCGA) [14]. The TCGA dataset included 37 normal mucosa samples and 203 tumor samples, 25 of which carry a *BRAF* mutation and 81 a *KRAS* mutation.

Initial testing was performed to confirm that the DNA methylation levels observed in the cell lines are comparable to those from the TCGA dataset. We compared the average β-value of all probes for each sample, and showed that our primary cell lines are comparable to the TCGA dataset, with levels of global DNA methylation similar to tumor samples. (S2 Fig, p = 0.23). To further confirm this we compared methylation levels which were grouped according to their CpG island annotations (displayed in S3 Fig). Probes annotated to CpG islands (including shores and shelves) showed hypermethylation in both the TCGA tumor samples and the cell line samples (p = 2.0×10^−3^ and p = 2.2×10^−5^ respectively). In line with hypomethylation of CpG sites in intergenic regions during tumorigenesis, in both TCGA tumors and cell line samples, probes outside CpG islands were found to be hypomethylated when compared to the TCGA-normal colon mucosa samples (p = 4.0×10^−15^ and p = 1.5×10^−13^ respectively). Interestingly, *BRAF* mutant CRC showed higher DNA methylation levels in all probe catagories, also probes outside CpG islands, suggesting that the intergenic hypomethylation is lower in *BRAF* mutant CRC than in *BRAF* wildtype CRC (S3 Fig).

### Global DNA hypomethylation

Comparison of the average β-values of the cell line samples showed that JVE241 deviated consistently from the other cell lines. Upon closer inspection, we noticed that the average β-value of probes within CpG islands was within the range for normal (TCGA) colon mucosa, but the CpGI shores, shelves and non-CpGI related probes were all found to be hypomethylated. Interestingly, one of the TCGA samples (TCGA-DM-A28E-01) also showed a similar pattern of extensive genome-wide hypomethylation without CpGI hypermethylation. Whole exome sequencing was performed by the TCGA, which revealed no mutations in any DNA-methylation related genes such as the DNMT or TET-enzymes.

DNA methylation is known to be important for genomic stability, and hypomethylation has been reported to correlate with genomic instability [22]. Surprisingly, the two hypomethylated samples, JVE241 and TCGA-DM-A28E-01 showed no evidence of increased genomic instability or chromotrypsis in the copy number profile we generated using the HumanExome12v1 and HumanMethylation450 data respectively (S4 Fig).

### Validation in TCGA data

To further investigate our results extracted the methylation values of the 706 loci from the TCGA dataset and compared the *BRAF*-mutant samples with the *KRAS*-mutant and WT samples. The average methylation value of the 706 loci is lower in the TCGA data than in the cell lines. This is a result of the higher percentage of *BRAF*-mutant samples in the cell line dataset (29% against 12%). The average difference in methylation of *BRAF*-mutant samples compared to *KRAS*-mutant and WT samples showed a strong correlation (r = 0.7911). This confirms the BRAF-mutation associated hypermethylation discovered in the cell lines.

We then decided to focus on the 20 loci identified to show a correlation between DNA methylation and transcriptional downregulation, which all showed significant hypermethylation in *BRAF* mutant samples compared to the *KRAS*-mutant and WT samples (S2 Table). Subsequently, the DNA methylation results were combined with the RSEM gene expression values downloaded through http://www.cbioportal.org/ [23;24]. For 10 of the genes the RSEM expression level was below 10 in over 25% of the samples. These genes were excluded from further analyses as we consider these genes to be extremely lowly expressed (S2 Table). This set of genes included amongst others *ST8SIA1* and *LRAT*, which have also been shown to be hypermethylated in CRC [18;20]. The remaining 10 genes include *CD1D*, *MEIS1* and *STC2*. Downregulation of *STC2* has been reported previously in CRC as a result of promoter hypermethylation [21]. *MEIS1* promoter hypermethylation has also previously been described to result in transcriptional repression, specifically in *BRAF* mutant CRC [19].

### CD1D

*CD1D* is an MHC-like molecule involved in antigen presentation of lipids and glycoproteins. Recently Ni et al. showed that cytotoxic activity of invariant-NKT cells against CRC cell lines is enhanced by *CD1D* upregulation [25]. Downregulation of *CD1D* as a result of DNA hypermethylation in tumors carrying a *BRAF* mutation could therefore be a crucial step in the evasion of the immune system.

First *CD1D* expression and methylation was validated by qRT-PCR and bisulfite sequencing analysis (BSA) in the 21 cell lines. The qRT-PCR results strongly correlated to the expression data obtained from the array-based expression dataset (pearson’s r = 0.858, p < 1×10^−5^). Similarly, BSA showed a strong correlation with the array data (pearson’s r = 0.9657, p < 1.0×10^−5^).

**Fig 4:**
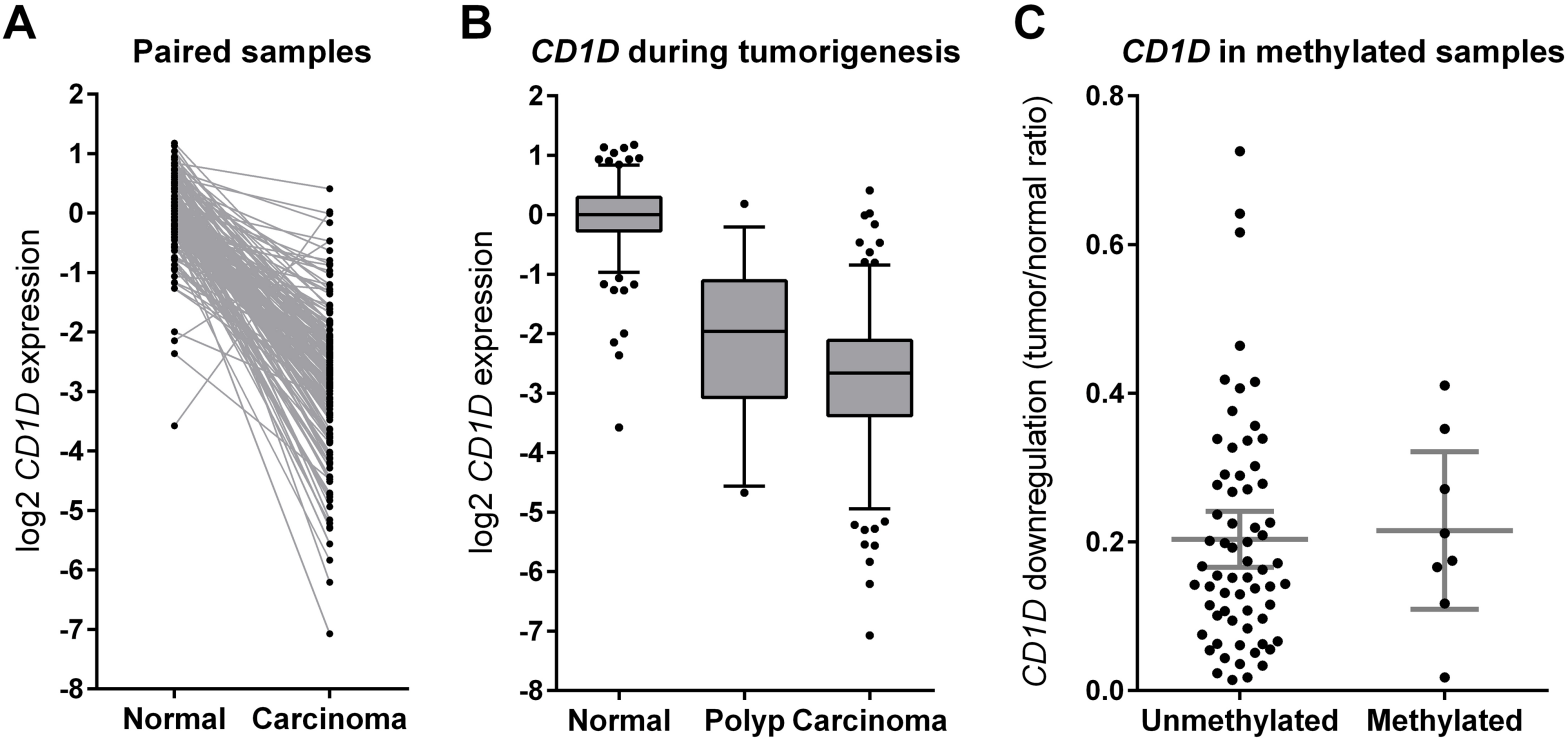
*CD1D* expression analyses in patient samples. qRT-PCR expression analysis of *CD1D* in 184 paired carcinoma and normal samples. A strong transcriptional downregulation was observed in the carcinoma compared to the normal samples. Gene expression values are relative to the median of the normal samples (4A). Comparison of *CD1D* expression in 34 polyp samples compared to the 184 normal and carcinoma samples. *CD1D* is also downregulated in the polyps, but downregulation in polyps is not as strong as in the carcinoma. Gene expression values are relative to the median of the normal samples. The box represent the 25-75-quartiles, the whiskers represent the 5–95% percentiles (4B). Comparison of the *CD1D* expression normal/carcinoma ratio in methylated versus unmethylated carcinoma. *CD1D* hypermethylation does not affect the degree of transcriptional downregulation. The bars represent the mean with 95% confidence interval (4C).

We investigated gene expression levels of *CD1D* in a set of 184 paired normal and carcinoma samples and 34 polyp samples of which cDNA from fresh-frozen tissue was available. *CD1D* expression was lower in nearly all carcinoma samples when compared to adjacent normal mucosa (student’s T-test, P = 1.4×10^−67^, Fig 4A). Also the polyp samples showed transcriptional downregulation of *CD1D*, albeit less than the carcinoma samples (student’s T-test, P = 1.2×10^−3^, Fig 4B), indicating that *CD1D* downregulation occurs early during tumorigenesis.

We show that the hypermethylation does not result in lower *CD1D* expression than in unmethylated carcinoma (Fig 4C). BSA of 73 carcinoma samples identified 8 hypermethylated samples, which did not show a stronger transcriptional downregulation than the unmethylated samples.

Ni et al. showed that upregulation of *CD1D* can enhance anti-tumor activity of invariant natural killer cells *in vitro*. Interestingly, increased signalling through the MAPK-pathway was shown to increase *CD1D* expression. These experiments were performed in HT29 which is unmethylated at the *CD1D* locus as tested using with our BSA. Our results showed that *CD1D* expression is lower in all CRC, regardless of *CD1D* hypermethylation. We now conclude that the high expression observed in the unmethylated cell line samples is due to upregulation occurring *in vitro*. Possibly this transcriptional upregulation would take place in all cell lines, were it not that some of the cell lines have *CD1D* hypermethylation. We cannot exclude the possibility that *CD1D* upregulation also occurs in tumors *in vivo*, but this would not be a growth advantage, as these cells would be targeted by the immune system. This would explain why no carcinoma samples are found with high *CD1D* expression, neither in the TCGA dataset, nor in our patient cohort.

### Correlation between CpGI hypermethylation and expression in TCGA samples

To visualize the correlation between gene expression and DNA hypermethylation for 10 remaining genes, we plotted the RSEM expression values against the average β-value for the genes found to be expressed in the TCGA dataset. The scatter plots are included in S6 Fig, correlation coefficients are listed in S2 Table. Similar to our findings in our patient samples, *CD1D* expression did not correlate with DNA methylation in the TCGA dataset (r = -0.1937, p > 0.1).

**Fig 5:**
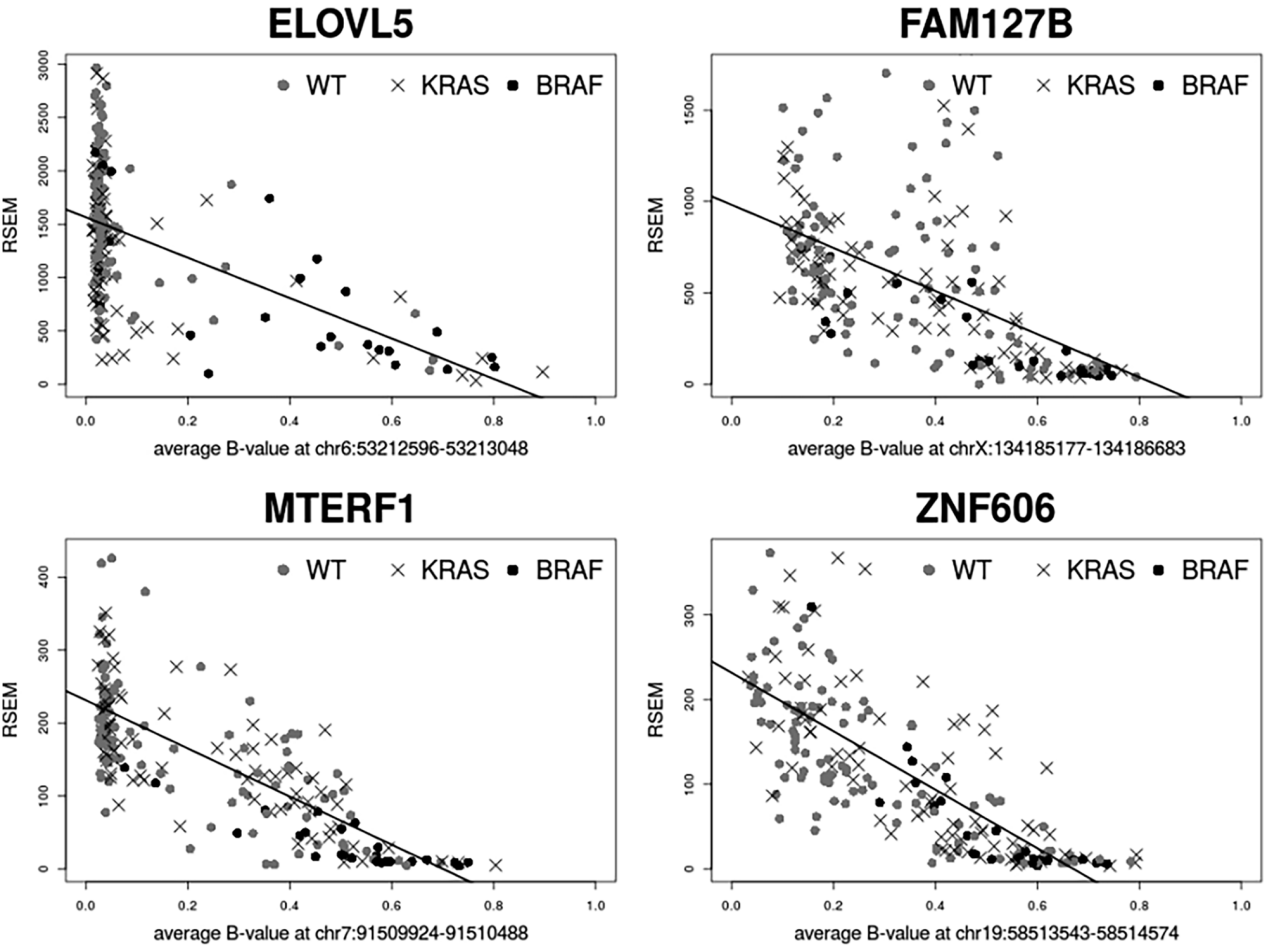
Genes showing correlation between expression and methylation in the TCGA data. RSEM gene expression values were plotted against the average β-values of the methylation loci.

We found that *ELOVL5*, *FAM127B*, *MTERF1* and *ZNF606* all show a negative correlation with gene expression in the TCGA data smaller than -0.50 (Fig 5, S2 Table). To further explore the deregulation of these 4 genes during CRC tumorigenesis, we consulted the GENT gene expression database [26]. We found that *FAM127B*, *MTERF1* and *ZNF606* are downregulated in the majority of tumors (S7A Fig). Contrastingly, *ELOVL5* was upregulated during tumorigenesis, suggesting importance of this gene in CRC, therefore we continued analysis of *ELOVL5*. We performed qRT-PCR in our paired normal and CRC samples, confirming upregulation of *ELOVL5* in CRC (p = 8.9×10^−10^ S7B Fig).

### Association of *ELOVL5* DNA methylation and protein levels with overall survival

**Fig 6:**
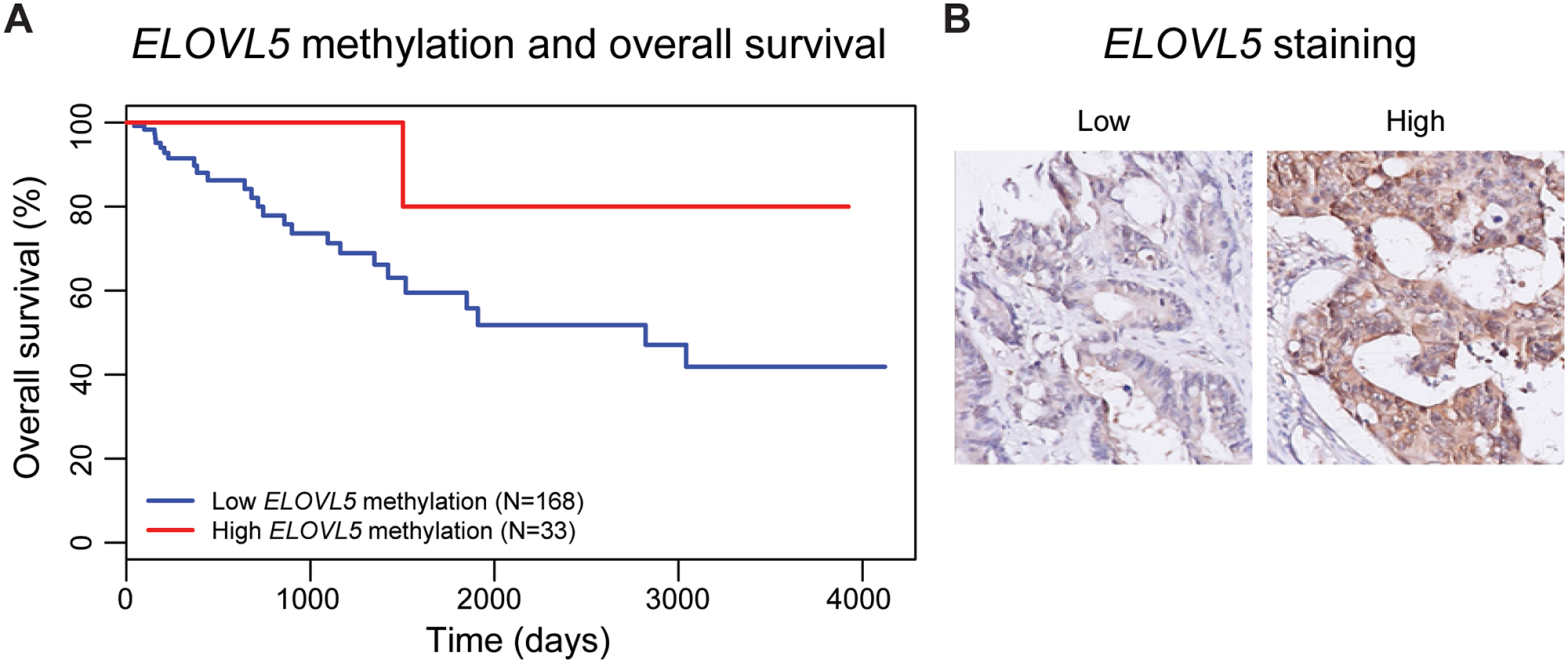
Further investigation of ELOVL5. Suggestive protective effect of promoter DNA hypermethylation of *ELOVL5* (6A, p = 0.07, log-rank test). Examples of tumors with low and high staining for ELOVL5 in neoplastic cells (6B).

To further investigate whether *ELOVL5* methylation associated with survival outcome of CRC, we performed survival analysis on the TCGA data. We divided the samples into 2 groups, with either no methylation (β < 0.2) or methylated (β > 0.2) at *ELOVL5*. We observed a protective effect of *ELOVL5* hypermethylation on overall survival, but this was not statistically significant (Fig 6A, p = 0.07, log-rank test).

Additionally, we performed immunohistochemical staining of ELOVL5 in a cohort of 263 CRC samples. ELOVL5 staining was scored as low, medium or high, based on staining intensity in neoplastic cells. Samples with varying intensity of ELOVL5 staining were excluded from the analysis. Fig 6B shows example staining in CRC tumors with low and high ELOVL5 staining. The staining results are shown in Table 1. No relation was observed between ELOVL5 staining intensity of neoplastic cells and tumor stage, TNM staging or relapse-free-survival.

**Table 1:** ELOVL5 immunohistochemical staining results.

	ELOVL5 low	ELOVL5 medium	ELOVL5 high	Overall
**Number of patients**	76	121	66	263
**Gender (Male)**	37 (49%)	58 (48%)	35 (53%)	130 (49%)
**Age**	67	68	67	67
**Relapse-free-survival (years)**	4.11	3.7	3.8	3.8
**TNM stadium (AJCC 5)**				
Stage I	14 (33%)	17 (40%)	12 (28%)	43 (16%)
Stage II	29 (28%)	42 (41%)	31 (30%)	102 (39%)
Stage III	24 (35%)	32 (47%)	12 (18%)	68 (26%)
Stage IV	13(26%)	28 (56%)	9 (18%)	50 (19%)
**T-score**				
T1	9 (50%)	6 (33%)	3 (27%)	18 (7%)
T2	9 (28%)	13 (40%)	10 (31%)	32 (12%)
T3	47 (30%)	75 (47%)	36 (23%)	158 (60%)
T4	15 (27%)	25 (45%)	15 (27%)	55 (21%)
**N-score**				
N0	37 (29%)	51 (40%)	38 (30%)	126 (53%)
N1	25 (37%)	33 (49%)	9 (13%)	67 (28%)
N2	10 (23%)	21 (48%)	12 (28%)	43 (18%)
**M-score**				
M0	53 (28%)	83 (44%)	52 (28%)	188 (85%)
M1	8 (24%)	20 (57%)	6 (17%)	34 (15%)

## Discussion

In this study we characterized 21 low passage CRC cell lines on DNA methylation level. Our analysis revealed that *BRAF*-mutant cell lines form a distinct group, therefore we chose to analyse a set of loci for which methylation patterns were previously associated with *BRAF*-mutations. The original *BRAF*-associated methylation loci were identified in a set of 20 paired tumor and normal tissues using Agilent 244k human CpG island microarrays [15]. We could validate 47.9% of the loci in our dataset. Both discovery and validation sets are of relatively small size and the sample type are also very different. Moreover, the platforms used to generate both datasets differ in chemistry and design. The high concordance between discovery and validation datasets, despite these differences we have mentioned, suggests to us that the loci identified in this study are true *BRAF*-associated methylation loci.

Although the *BRAF*-mutation status lies at the base of the discovery of these genes, our results show that hypermethylation of these genes is not exclusive to *BRAF*-mutant CRC. Instead we show that many of these loci also show hypermethylation in *KRAS*-mutant and WT CRC, making these results relevant for a larger subset of CRC. One of the genes that drew our interest was *CD1D*, an MHC-like molecule involved in antigen presentation of lipids and glycoproteins, which was recently shown to enhance cytotoxic activity of invariant-NKT cells against CRC cell lines [25]. Our validation in a large set of colorectal polyps and carcinomas showed *CD1D* downregulation occurs in nearly all CRC, regardless of DNA hypermethylation. We propose that like in nearly all tumors we tested, the tumors from which these cell lines were established also showed transcriptional downregulation of *CD1D*. The high expression of *CD1D* in the cell lines without *CD1D* promoter hypermethylation is most likely the result of upregulation during cell line establishment, and the DNA hypermethylation in some of the cell lines blocked this upregulation. *In vivo* such an upregulation would be disadvantageous for the tumor cells, as any cells in which *CD1D* upregulation occurs would be targeted by invariant-NKT cells, a selection pressure which is no longer present *in vitro*. Validation of these regions in the TCGA colon adenocarcinoma data identified a signature of 4 genes; *ELOVL5*, *FAM127B*, *MTERF1* and *ZNF606*, which are downregulated in hypermethylated tumors.

*ELOVL5* encodes Fatty acid elongase 5, which plays a crucial role in negative feedback regulation of lipogenesis [27]. Increases in lipogenesis have been reported to provide selective proliferation and survival in CRC cells [28]. Conversely, the survival analysis using TCGA data showed *ELOVL5* hypermethylation associated with improved overall survival. As *ELOVL5* methylation correlates with *MLH1* methylation, we postulate that this protective effect is related to hypermethylation of *MLH1*. *MLH1* hypermethylation strongly associates with microsattelite instability, a well known marker for good prognosis. Immunohistochemical staining of ELOVL5 in a series of 263 CRC tissues showed clear differences in protein levels between tumors, but we found no evidence for ELOVL5 protein level differences being associated with tumor stage or relapse free survival. We cannot exclude the possibility that *ELOVL5* methylation confers selective advantages to CRC cells, but to study this a large cohort of CRC would be required with information on DNA methylation status and survival and preferably also microsatellite instability status.

*MTERF1* encodes a mitochondrial protein with transcriptional termination activity. It binds to a 28 bp region within *tRNA*^*L1*^. Experiments have shown *MTERF1* only affects transcription on the light-strand, transcripts from the heavy-strand were unaffected. Experiments in mice have shown that the levels of 12S rRNA and 16S rRNA are increased after *MTERF1* knockdown [29]. 12S rRNA is essential for mitochondrial ribosome assembly, suggesting that *MTERF1* knockdown could potentially increase mitochondrial ribosome assembly, giving a growth advantage to the tumor cells [30]. Interestingly, the 16S rRNA has been found to encode a potential oncopeptide; Humanin, which has anti-apoptotic properties [31]. This suggests *MTERF1* downregulation is favourable for CRC cells, as it allows them to avoid apoptosis through Humanin overexpression.

*ZNF606* (*ZNF328*) encodes a 792 aminoacid protein with both a KRAB domain and a classical zinc-finger motif. The KRAB domain functions as a transcriptional repressor when in the vicinity of DNA, making *ZNF606* a transcriptional repressor. Experiments in COS-7 cells have shown that overexpression of *ZNF606* results in a reduction of SRE and AP-1 transcriptional activities. By inhibiting SRE and AP-1 signalling *ZNF606* serves as a negative regulator of the transcriptional activation downstream of the MAPK-pathway [32].

The function of *FAM127B* is unknown, therefore we cannot speculate as to whether the loss of *FAM127B* is beneficial to CRC cells. Overall, the transcriptional downregulation of *ELOVL5*, *MTERF1* and *ZNF606* shows potentially beneficial effect for the tumor cells. Together the loss of these 3 genes can increase lipogenesis, increase the downstream transcriptional effects of MAPK signalling and increase the level of anti-apoptotic protein Humanin.

## Supporting information

S1 Fig Variation of methylation loci. For all loci in the *BRAF*-associated methylation analysis of primary CRC cell lines, the standard deviation of the β-values for all cell lines was plotted against the average of the β-values for all cell lines. We found not-significant loci to be less variable between the cell lines (lower standard deviation). Most not-significant loci were either methylated in all cell lines or unmethylated in all cell lines. This excludes the possibility of these loci not being significant due to a cell line specific hypermethylation profile.
(TIF)

S2 Fig Average β-value per sample. The average β-value for all probes was calculated to visualize the global DNA methylation levels. The average β-value for the cell lines is comparable to that of the TCGA tumor samples. In red the average per group is displayed, with 95% confidence interval.
(TIF)

S3 Fig Average β-value on probe location relative to the nearest CpG island. Probes were grouped on their location relative to the nearest CpG island. The average β-value for the cell lines is comparable to that of the TCGA tumor samples. Both JVE241 and TCGA-DM-A28E-01 showed a very lower methylation level at non-CpG-island probes. In red the average per group is displayed, with 95% confidence interval.
(TIF)

S4 Fig Copy number alterations for TCGA-DM-A28E-01. Although this sample shows an extremely low DNA methylation level, there is no sign of severe chromosomal instability or chromotrypsis. This copy number profile was generated using the Infinium HumanMethylation450 data.
(TIF)

S5 Fig Comparison of DNA methylation levels in the cell lines and TCGA tumors for the tested loci. The average methylation level, rank in the analysis, BH-adjusted p-value and methylation difference between BRAF mutant and other samples was compared for the TCGA samples and the cell lines, showing good concordance between the cohorts.
(TIF)

S6 Fig Correlation between DNA methylation and gene expression in the TCGA samples for the 10 remaining genes. (PDF)

S7 Fig Gene expression of genes with strong correlation between expression and methylation. Gene expression values from the Gene Expression in Normal and Tumor database shows ELOVL5 is upregulated during tumorigenesis (**7A**) [26]. qRT-PCR analysis in our normal mucosa and paired CRC samples confirmed upregulation of *ELOVL5* in CRC. 22% of samples did not show upregulation of *ELOVL5* during tumorigenesis (**7B**).
(TIF)

S1 Table TCGA samples used in this study, with their *BRAF* and *KRAS* mutation status. (XLSX)

S2 Table Methylation and expression data for the top 20 genes identified in this study as being subject to *BRAF* mutation associated transcriptional downregulation through DNA hypermethylation. (XLSX)
