# Supplementary figures and images for: Methylation associated transcriptional repression of ELOVL5 in novel colorectal cancer cell lines
Source: PLoS One. 2017 Sep 20;12(9):e0184900. doi: 10.1371/journal.pone.0184900 (PMC5607170; doi:10.1371/journal.pone.0184900)

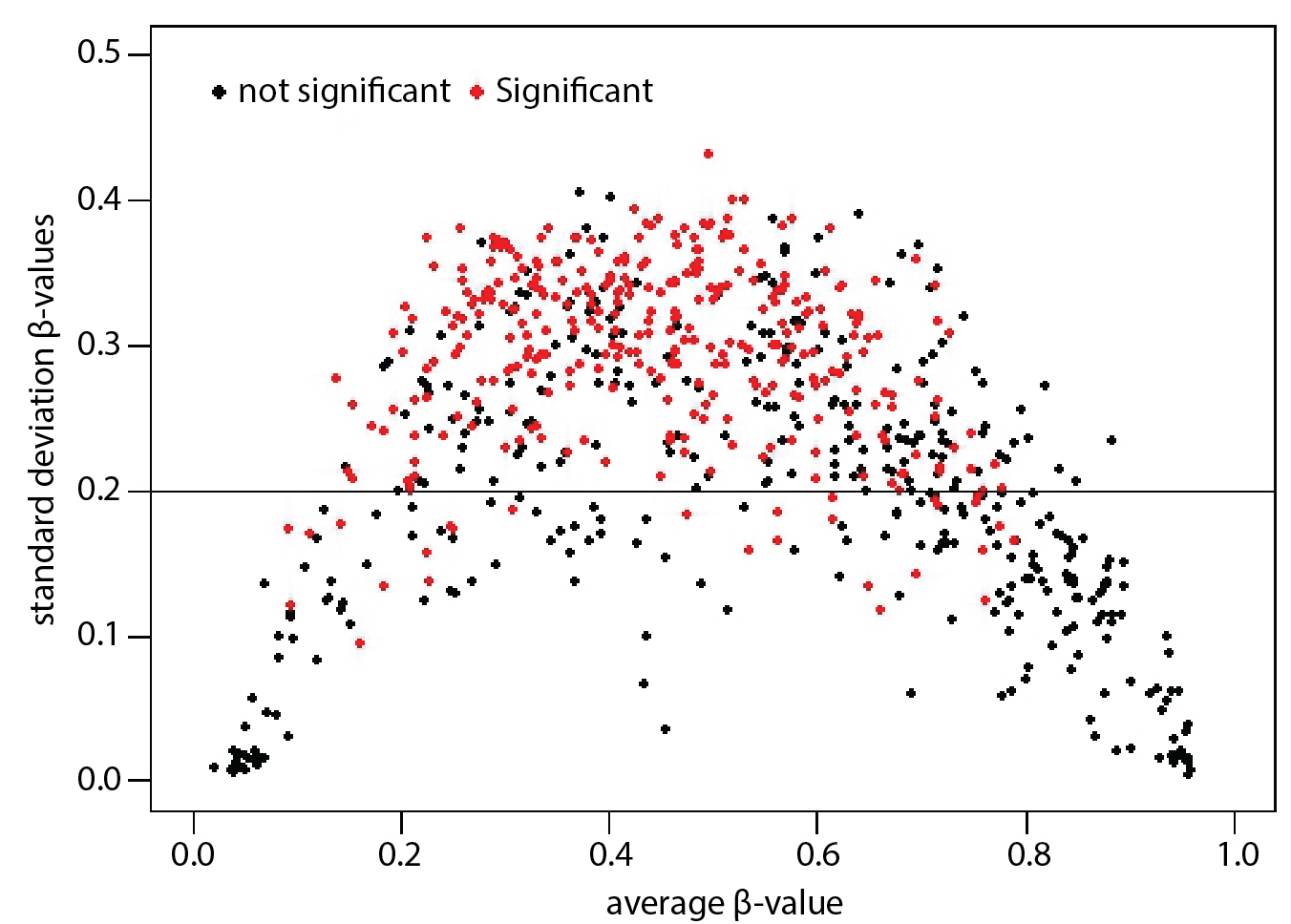

Supplement: S1 Fig — For all loci in the BRAF-associated methylation analysis of primary CRC cell lines, the standard deviation of the β-values for all cell lines was plotted against the average of the β-values for all cell lines. We found not-significant loci to be less variable between the cell lines (lower standard deviation). Most not-significant loci were either methylated in all cell lines or unmethylated in all cell lines. This excludes the possibility of these loci not being significant due to a cell line specific hypermethylation profile. (TIF) [file pone.0184900.s001.tif]

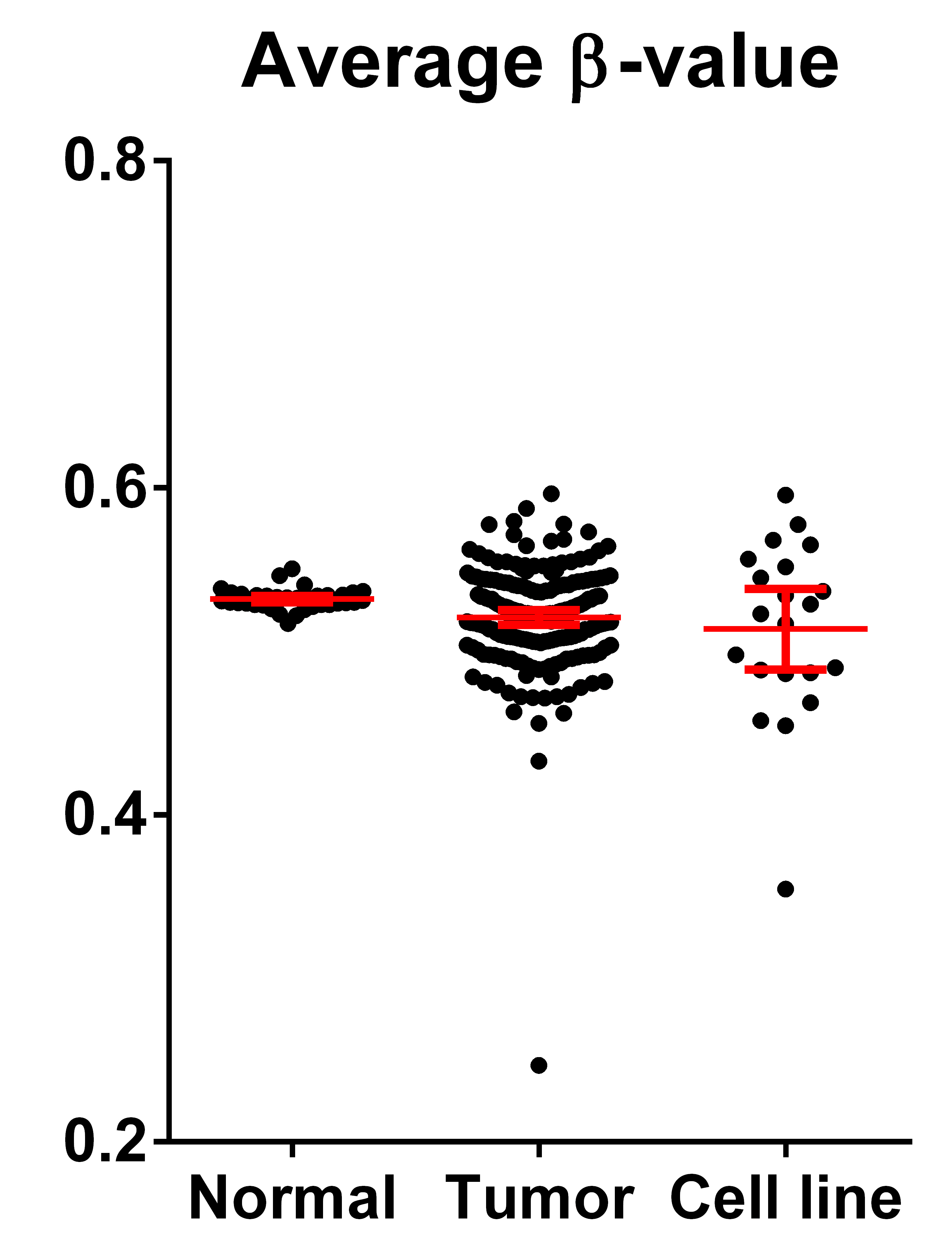

Supplement: S2 Fig — The average β-value for all probes was calculated to visualize the global DNA methylation levels. The average β-value for the cell lines is comparable to that of the TCGA tumor samples. In red the average per group is displayed, with 95% confidence interval. (TIF) [file pone.0184900.s002.tif]

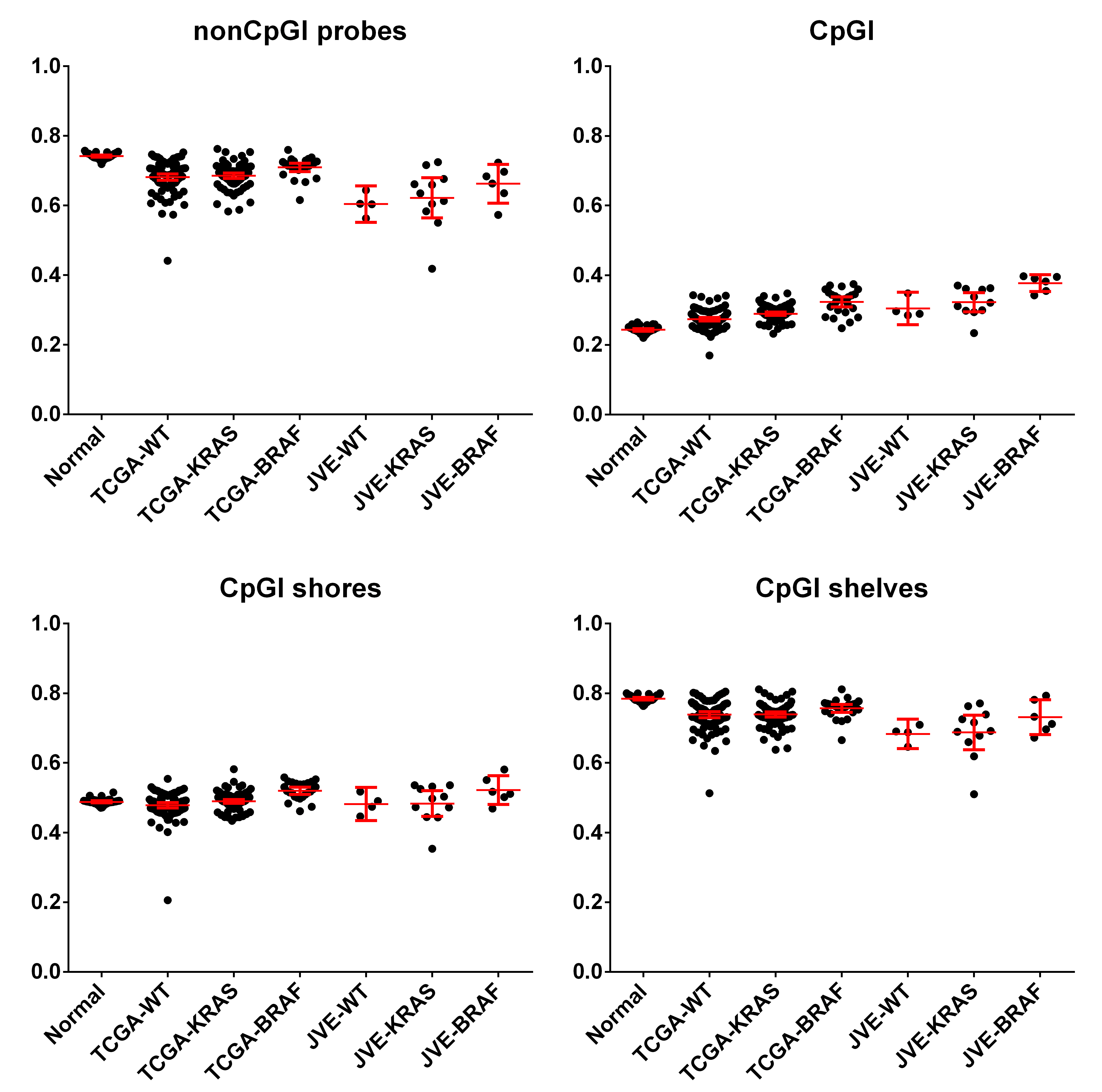

Supplement: S3 Fig — Probes were grouped on their location relative to the nearest CpG island. The average β-value for the cell lines is comparable to that of the TCGA tumor samples. Both JVE241 and TCGA-DM-A28E-01 showed a very lower methylation level at non-CpG-island probes. In red the average per group is displayed, with 95% confidence interval. (TIF) [file pone.0184900.s003.tif]

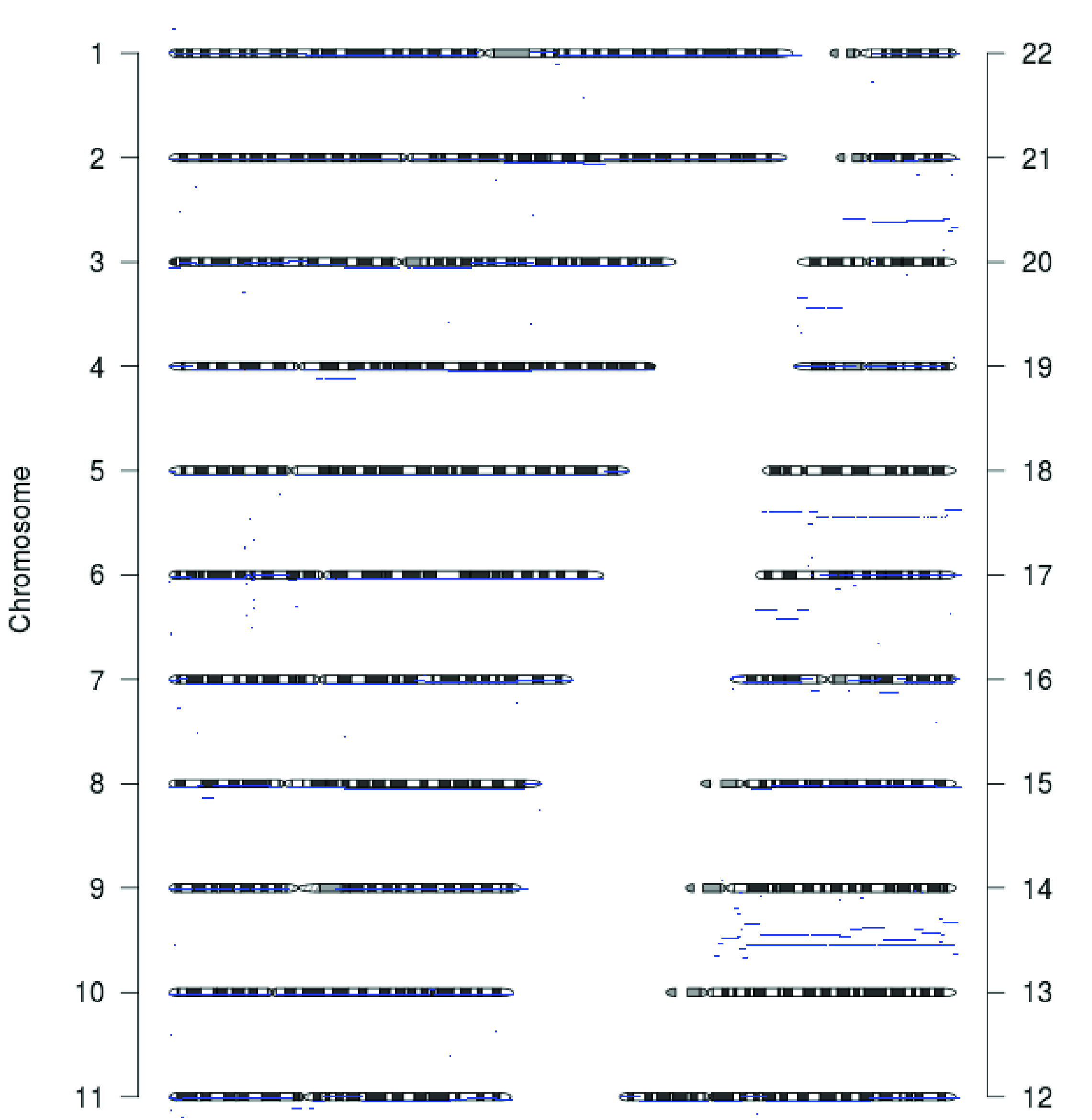

Supplement: S4 Fig — Although this sample shows an extremely low DNA methylation level, there is no sign of severe chromosomal instability or chromotrypsis. This copy number profile was generated using the Infinium HumanMethylation450 data. (TIF) [file pone.0184900.s004.tif]

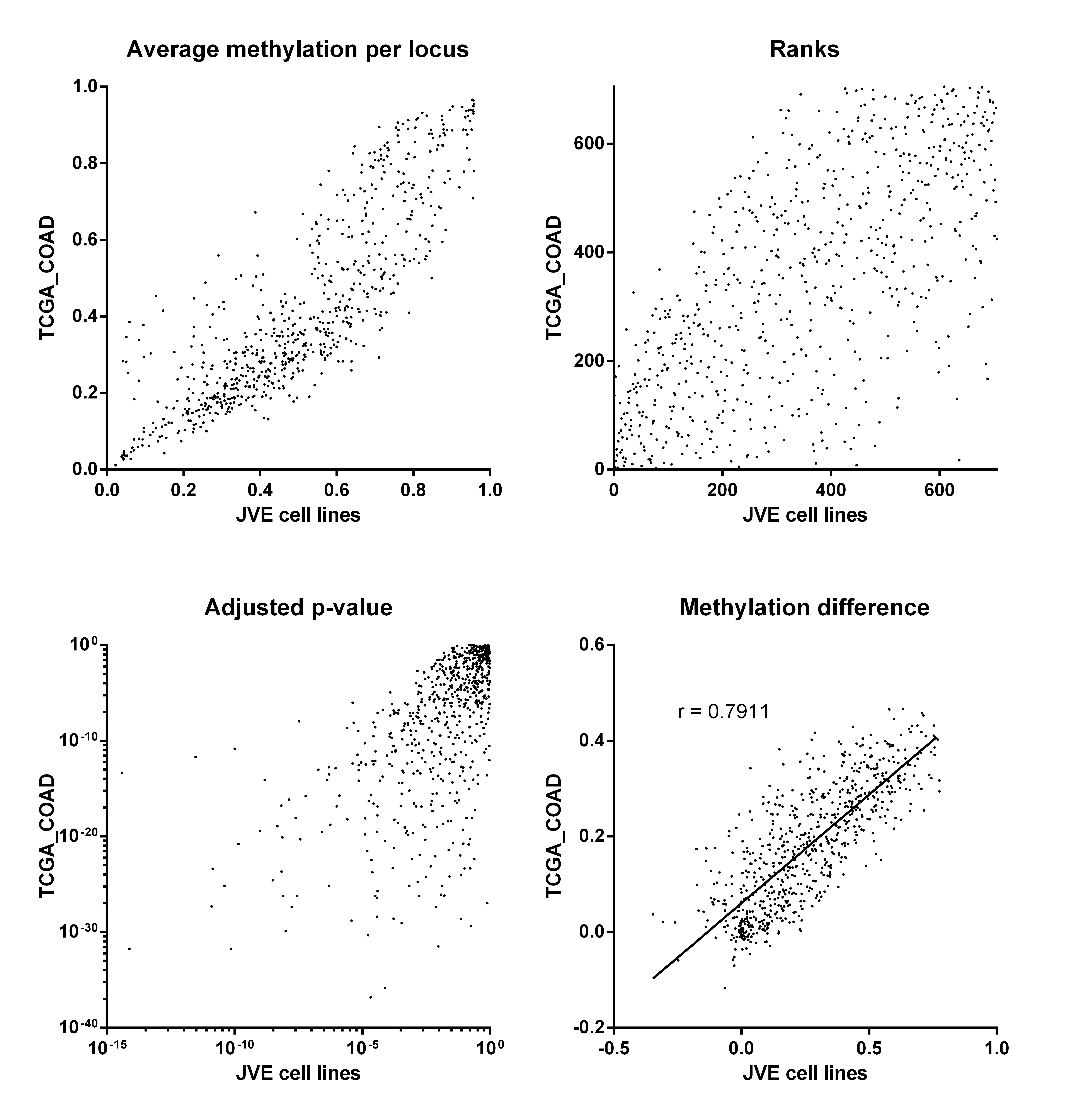

Supplement: S5 Fig — The average methylation level, rank in the analysis, BH-adjusted p-value and methylation difference between BRAF mutant and other samples was compared for the TCGA samples and the cell lines, showing good concordance between the cohorts. (TIF) [file pone.0184900.s005.tif]

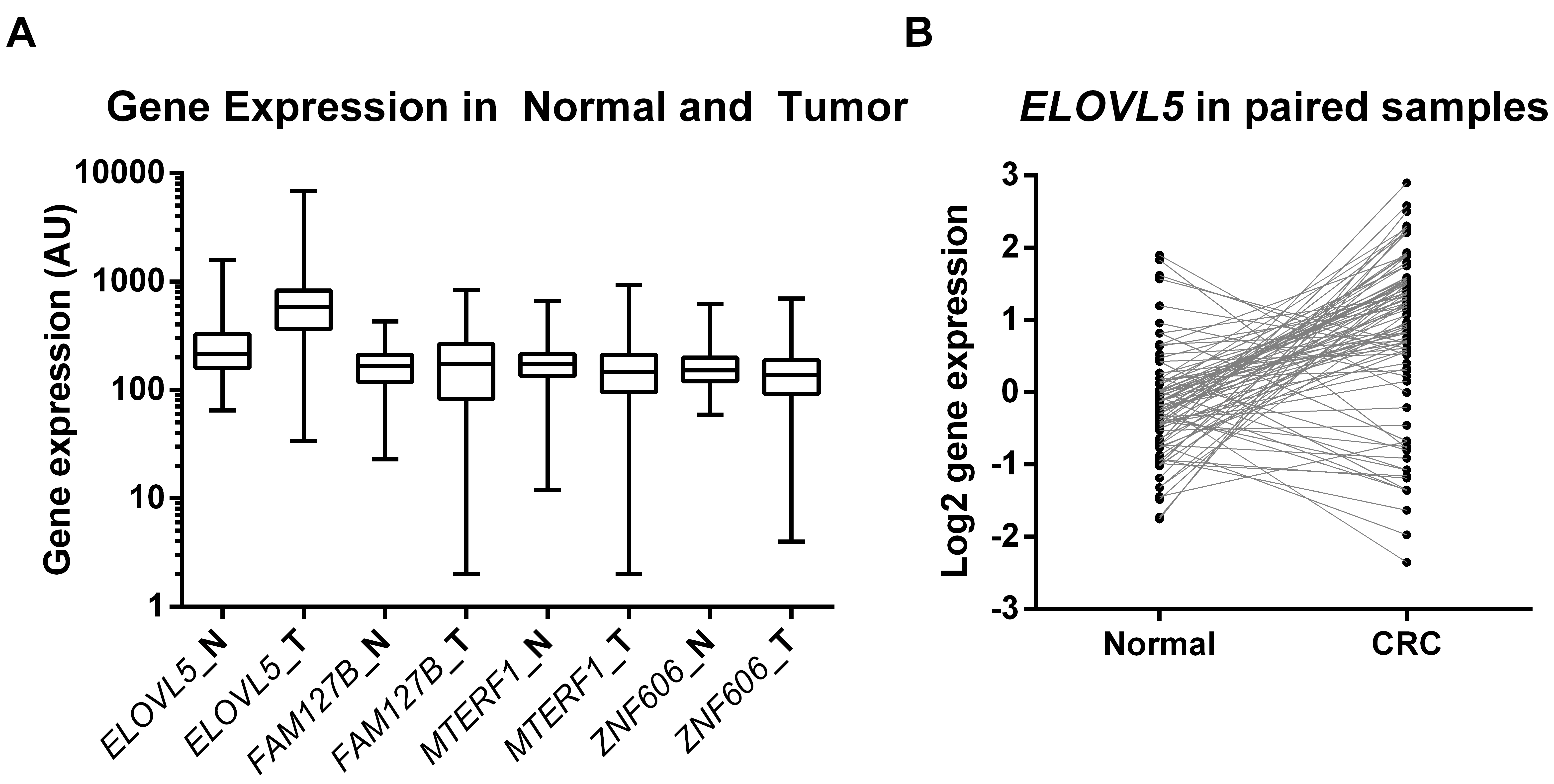

Supplement: S7 Fig — Gene expression values from the Gene Expression in Normal and Tumor database shows ELOVL5 is upregulated during tumorigenesis (7A) [26]. qRT-PCR analysis in our normal mucosa and paired CRC samples confirmed upregulation of ELOVL5 in CRC. 22% of samples did not show upregulation of ELOVL5 during tumorigenesis (7B). (TIF) [file pone.0184900.s007.tif]
