# Supplementary material for: Methylation associated transcriptional repression of ELOVL5 in novel colorectal cancer cell lines
Source: PLoS One. 2017 Sep 20;12(9):e0184900. doi: 10.1371/journal.pone.0184900 (PMC5607170; doi:10.1371/journal.pone.0184900)
Supplement: S6 Fig — (PDF) [file pone.0184900.s006.pdf]

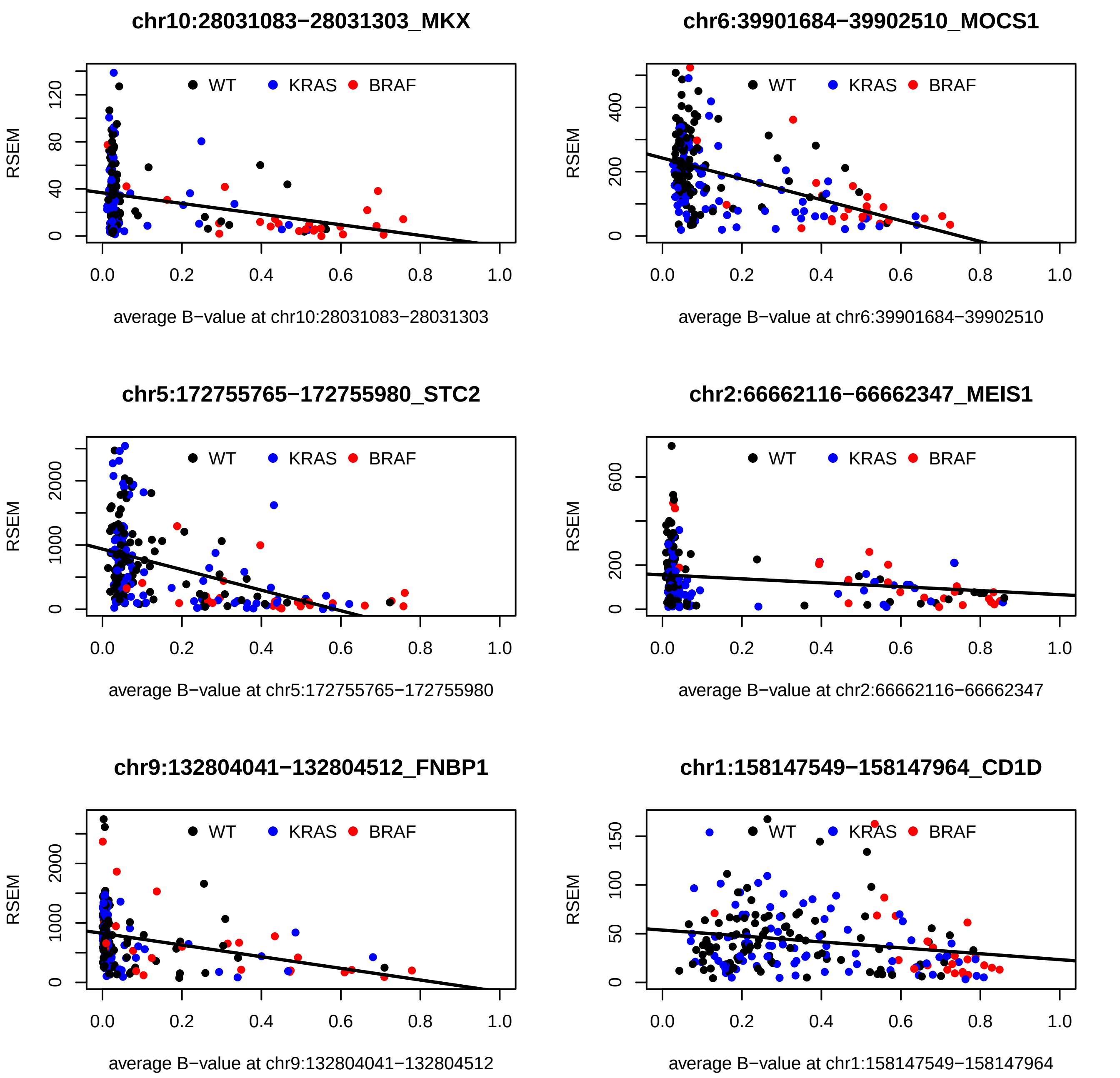

chr10:28031083−28031303_MKX
WT
KRAS
BRAF
RSEM
0
40
80
120
0.0
0.2
0.4
0.6
0.8
1.0
average B−value at chr10:28031083−28031303
chr6:39901684−39902510_MOCS1
WT
KRAS
BRAF
RSEM
0
200
400
0.0
0.2
0.4
0.6
0.8
1.0
average B−value at chr6:39901684−39902510
chr5:172755765−172755980_STC2
WT
KRAS
BRAF
RSEM
0
1000
2000
0.0
0.2
0.4
0.6
0.8
1.0
average B−value at chr5:172755765−172755980
chr2:66662116−66662347_MEIS1
WT
KRAS
BRAF
RSEM
0
200
600
0.0
0.2
0.4
0.6
0.8
1.0
average B−value at chr2:66662116−66662347
chr9:132804041−132804512_FNBP1
WT
KRAS
BRAF
RSEM
0
1000
2000
0.0
0.2
0.4
0.6
0.8
1.0
average B−value at chr9:132804041−132804512
chr1:158147549−158147964_CD1D
WT
KRAS
BRAF
RSEM
0
50
100
150
0.0
0.2
0.4
0.6
0.8
1.0
average B−value at chr1:158147549−158147964

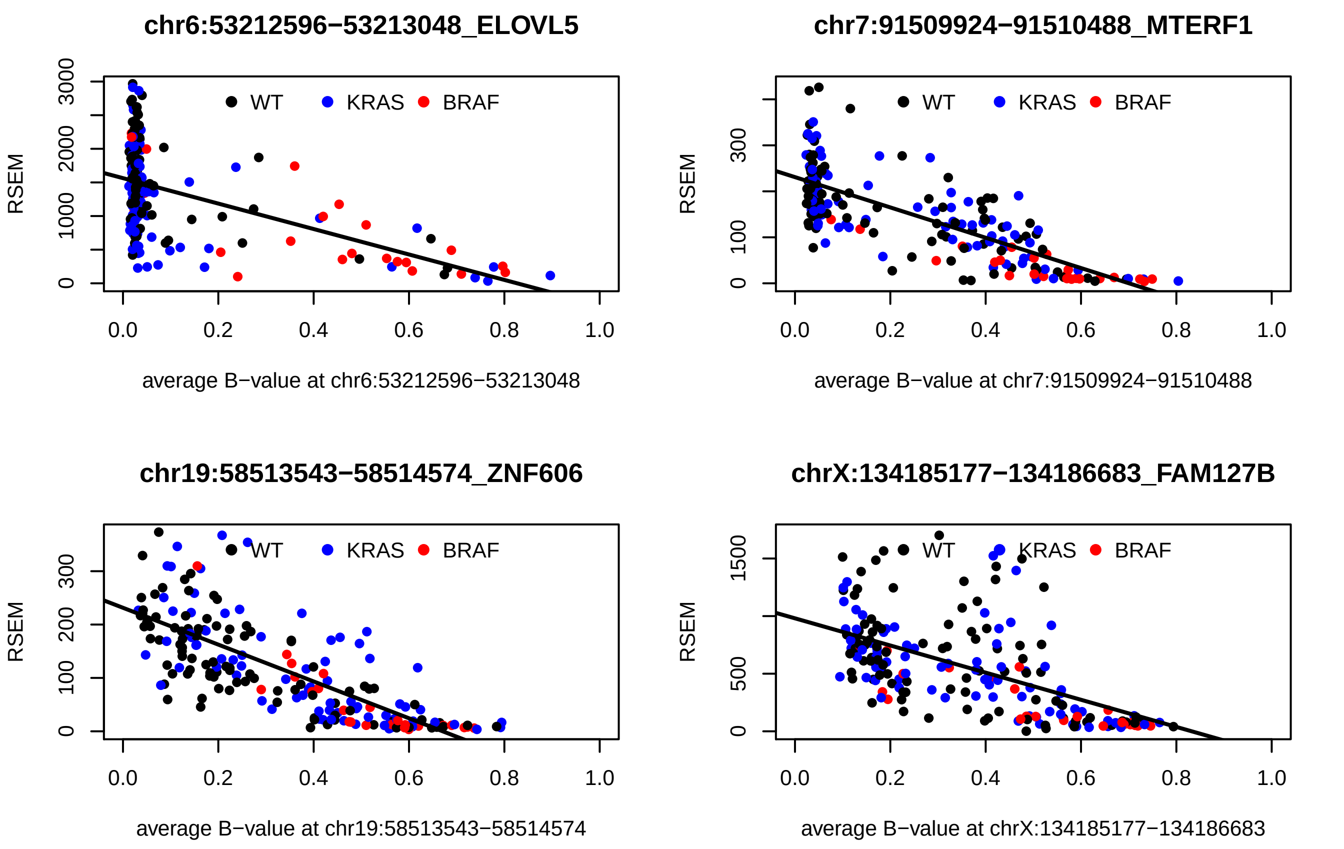
chr6:53212596−53213048_ELOVL5
WT KRAS BRAF
RSEM
0 1000 2000 3000
0.0 0.2 0.4 0.6 0.8 1.0
average B−value at chr6:53212596−53213048
chr7:91509924−91510488_MTERF1
WT KRAS BRAF
RSEM
0 100 300
0.0 0.2 0.4 0.6 0.8 1.0
average B−value at chr7:91509924−91510488
chr19:58513543−58514574_ZNF606
WT KRAS BRAF
RSEM
0 100 200 300
0.0 0.2 0.4 0.6 0.8 1.0
average B−value at chr19:58513543−58514574
chrX:134185177−134186683_FAM127B
WT KRAS BRAF
RSEM
0 500 1500
0.0 0.2 0.4 0.6 0.8 1.0
average B−value at chrX:134185177−134186683
